# Molecular detection of human T-lymphotropic virus type 1 infection among oncology patients in Germany: A retrospective view

**DOI:** 10.1371/journal.pone.0217560

**Published:** 2019-05-28

**Authors:** Matias Ruggieri, Carolina Berini, Nicolas Ducasa, Miroslav Malkovsky, Paul Fisch, Mirna Biglione

**Affiliations:** 1 Institut für Klinische Pathologie, Universitätsklinikum Freiburg, Freiburg, Germany; 2 Instituto de Investigaciones Biomédicas en Retrovirus y SIDA (INBIRS), CONICET-Universidad de Buenos Aires, Buenos Aires, Argentina; 3 UW School of Medicine and Public Health, Madison, Wisconsin, United States of America; Institute of Tropical Medicine Antwerp, BELGIUM

## Abstract

Human T-cell lymphotropic virus (HTLV) belongs to a larger group of primate T-cell lymphotropic viruses (PTLVs) within the family Retroviridae. It is estimated that 10 to 20 million people worldwide may be infected with HTLV-1. Although most of them are asymptomatic, around 5% of infected individuals may develop either HTLV-1 Associated Myelopathy/Tropical Spastic Paraparesis (HAM/TSP) or Adult T-cell Leukaemia/Lymphoma (ATLL). Public Health authorities in many countries have implemented routine blood-donor tests for HTLV-specific antibodies; but this is not the case for Germany since the reported prevalence is very low (7/100,000). With the aim to evaluate retrospectively the presence of HTLV-1 among oncology patients in this country, samples stored at the Universitätsklinikum Freiburg, were analyzed. For this purpose, two different nested-PCR (n-PCR) protocols have been modified and set up for HTLV-1 detection. One positive case was detected by n-PCR among 406 samples (0,25%) in a period of 5 years (2008–2012) corresponding to a T-Cell Lymphoma. Despite the low prevalence, this virus is circulating in Germany, probably due to the increasing numbers of immigrants in these last years. Physicians should consider HTLV-1 infection and suspect it taking in account the ethnic and relation to endemic regions regardless the patient's residence.

## Introduction

Human T-cell lymphotropic viruses (HTLVs) belong to a larger group of primate T-cell lymphotropic viruses (PTLVs) within the family Retroviridae. To date, four different types have been identified: HTLV types 1, 2, 3 and 4 [1]. HTLV-1 and 2 are classified into the genus Deltaretrovirus. Transmission of HTLV-1 can occur through sexual contact, blood transfusion, organ donation, sharing injecting equipment, as well as from mother-to-child (mainly through breast-feeding) [2–4].

HTLV-1 was the first human retrovirus to be isolated from a patient with a T-cell malignancy in 1979 [5]. It is estimated that 10 to 20 million people worldwide may be infected with HTLV-1. The main HTLV-1 highly endemic regions are the Southwestern part of Japan, sub-Saharan Africa and South America, the Caribbean area, and foci in Middle East and Australo-Melanesia [6].

HTLV-1 is the causative agent of serious illnesses with unsatisfactory treatment options, including inflammatory syndromes such as HTLV-1-Associated Myelopathy/Tropical Spastic Paraparesis (HAM/TSP), uveitis, Adult T-cell Leukaemia/Lymphoma (ATLL), opportunistic infections such as Strongyloides stercoralis hyperinfection and a range of lung diseases, including bronchiectasis, that is causing high mortality in Central Northern Australia [7] among others. Although most of the HTLV-1 carriers are asymptomatic, the life time risk of developing one of the above-mentioned disorders is estimated to be around 5%, depending on the sex and the geographic area [8, 9]

Several South American countries have naturally occurring endemic regions of HTLV-1, while most European countries present low prevalence (0.001 ± 0.03%), with Romania being the only endemic country for HTLV-1 infection in this continent. It is believed that over 80% of infected people in Europe are originally from endemic regions such as West Indies (Caribbean), Africa and South America [6, 10]. HTLV-1 is also prevalent (from 3–25%) among poly-transfused patients and at-risk populations [11, 12].

Various Public Health authorities in many countries have implemented routine blood-donor tests for HTLV-specific antibodies; the screening for HTLV-1/2 is mandatory in Japan, Canada, the Caribbean and some South America countries. In Europe, it is mandatory in several countries such as UK, France, Demark, Greece, Portugal, Romania and Sweden; and recommended in others such as Ireland, Luxembourg and Norway. In contrast, for example, in Italy and Germany, the HTLV-1/2 blood screening is not even recommended [13]. Furthermore, on individuals who donated organs, the screening of HTLV-1 is only mandatory in few European countries such as the UK and France, although there is a very high rate of myelopathy that follows infection in the context of solid organ transplantation [14]. Nevertheless, the European Union establishes in cases of cell and/or tissue donations that HTLV-1 antibody testing must be performed for donors living in, or originating from high-incidence areas or with sexual partners originating from those areas or when the donor’s parents originate from those areas [15].

Considering that the routine screening for HTLV-1/2 cannot distinguish between these two viruses, repeatedly reactive samples by screening tests are further tested by Western blot (WB), which may allow to ascertain the specific HTLV-type-seropositivity or -seronegativity. Nevertheless, the indeterminate serological results by WB are a frequent problem in HTLV-1 testing and this often leads to difficulties in the interpretation and counseling [16, 17]. One possible solution to this problem is to amplify conserved segments of the provirus using a n-PCR, which has a very high (practically 100%) sensitivity and can detect one MT2 cell (8 proviral copies)/1x10^6^ non-infected cells [18, 19].

The focus of this study was to evaluate retrospectively the presence of HTLV-1 in oncology samples stored at the Universitätsklinikum Freiburg, Germany. For this purpose, two different n-PCR protocols have been modified and set up for HTLV-1 detection.

## Materials and methods

### Samples studied

A blind study was conducted. A total of 410 paraffined biopsies and/or blood samples stored in the Pathology Department at Freiburg Uniklinik, Germany, during the period 2008–2012, with a presumptive diagnosis of malignancy were selected and analyzed for HTLV-1. To set up the ideal n-PCR conditions, the HTLV-1-producing cell line MT2 and 6 samples from the INBIRS Institute in Argentina were used: 2 samples from 2 healthy individuals and 4 samples from 4 HTLV-1-positive individuals (one from an asymptomatic case, one from an ATLL patient and 2 from patients with HAM/TSP).

### DNA extraction

DNA was extracted from tissue samples in paraffin using the DNA FFPE Tissue Kit (Qiagen, Hilden, Germany). DNA from cell lines, peripheral blood mononuclear cells (PBMCs) or blood was obtained by column extraction (ADN PuriPrep-S kit, Highway, Inbio, Tandil, Argentina). For the HTLV-1-positive controls, DNA was extracted from a) 2 x 10^6^ MT2 cells or b) 2 x 10^6^ PBMCs from HTLV-1-infected persons or c) 200 μl of blood obtained from HTLV-1-infected persons. For the negative controls, DNA was extracted from the 2 healthy individuals’ cells (either 2 x 10^6^ PBMCs or 200 μl of blood).

### PCR amplification

#### n-PCR for a single sample

The first PCR amplification was performed in a 25 μl reaction volume containing Master Mix 1 (PCR Buffer 1x, Q solution 1x, MgCl_2_ 2,5 mM, dNTPs 0,2 mM, Primers 0,3 mM and Taq polymerase 0,5 units from Qiagen, Hilden, Germany), and 5 μl of DNA (20 ng/μl). The second round of PCR used 2 μl of the first round and the same Master Mix 2 (same as Master Mix 1 but CoralLoad PCR Buffer 1x instead of PCR Buffer 1x), with 10 mM of *pol* and *actin* primers, in a final volume of 25 μl (dx.doi.org/10.17504/protocols.io.zjyf4pw).

#### n-PCR for a pool of 10 samples

The first PCR amplification was performed in a 50 μl reaction volume containing Master Mix 1 with one unit of Taq DNA polymerase enzyme, and 2 μl of DNA (20 ng/μl) from either the MT2 cell line or from pooled DNA isolated from 10 different samples. The second round of the PCR was performed taking 5 μl of the first round and using the same amount of Master Mix 2 in a final volume of 25 μl (dx.doi.org/10.17504/protocols.io.2pygdpw).

#### Primers

Primers were chosen based on their location within the prototype HTLV-1 genomic sequence available in the GenBank J02029.1, considering conserved sequences, and designed to avoid complementary sequences and strong internal secondary structures. Outer *pol* primers PolEF (5´ TTTAGGTGCCCAAACTGGAG 3´) and PolER (5´GCAGGATATTGGAAGCCTCAG3´) only anneal with HTLV-1 virus sequence. As inner primers PolIF (5´GCCCTCATGCCAGTGTTTAC3´) and PolIR (5´CCTGGAGATGGGATCAGGTAG3´) were selected. The forward (5´ATCGAGCACGGCATCGTCACCAAC3´) and reverse (5´GTTGAAGGTCTCAAACATGATCTG3´) *actin* primers were designed based on their location in the sequence GenBank NG_006672.1.

**PCR cycles**. Cycling conditions on a TProfessional TRIO Thermocycler (Biometra, California, USA) were 10 sec 94°C, 20 sec 54°C, 45 sec 70°C for the first round and 10 sec 94°C, 20 sec 52°C, 45 sec 70°C for the second round. Both PCRs were amplified for 40 cycles. Before the cycling started, the PCR tubes were heated to 94°C for 4 min. After the last cycle, they were heated to 70°C for 5 min and then kept at 4°C. Amplified products were loaded onto a 2% agarose gel stained with Gel Red Nucleic Acid Gel Stain (Biotium, Jena, Germany). Samples were run at 110 mV for 50 min. Bands were observed under ultraviolet (UV) luminometer (Fusion-FX7-826.WL, Eberhardzell, Germany). The 50 and 100 bp DNA Ladders (Thermo Fisher Scientific, Germany) were used.

## Results

### Multiplex n-PCR

**Fig 1:**
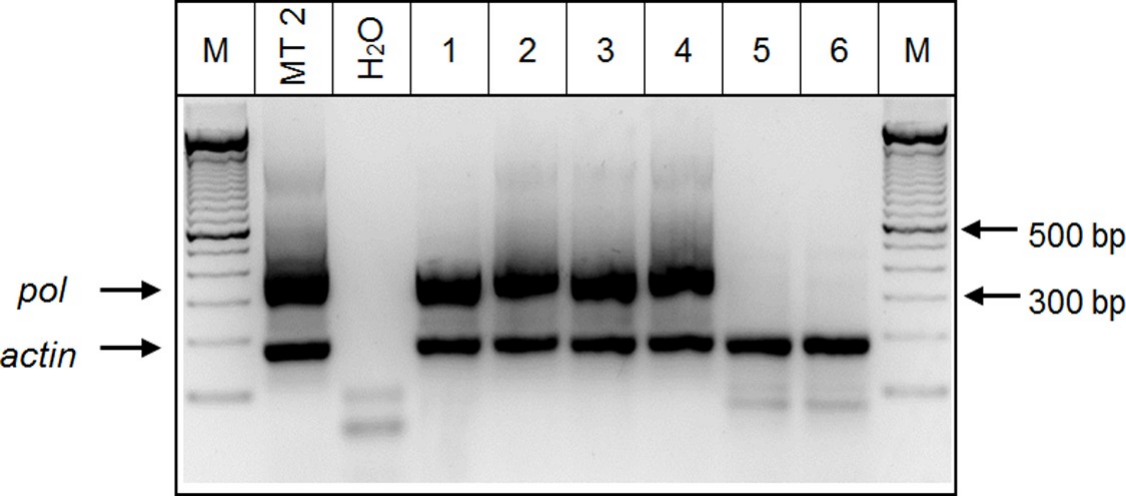
n-PCR with *pol* HTLV-1/actin co-amplification. MT2 (positive control), H_2_O (negative control), line 1: HAM/TSP sample, 2: ATLL sample, 3: HAM/TSP sample, 4: HTLV-1 asymptomatic sample, 5 and 6 samples from healthy blood donors. The arrows point to the bands of *pol* and *actin* amplification and 100 bp DNA Ladder (M).

The n-PCR of the HTLV-1 *pol* gene with the co-amplification of *actin* gene were combined to confirm the presence of DNA in the sample. Amplification of *pol* and *actin* genes were observed in the control sample (MT2) and the HTLV-1 positive samples (lines 1–4). Only *actin* gene amplified in the healthy blood donors, lines 5 and 6 (Fig 1).

**Fig 2:**
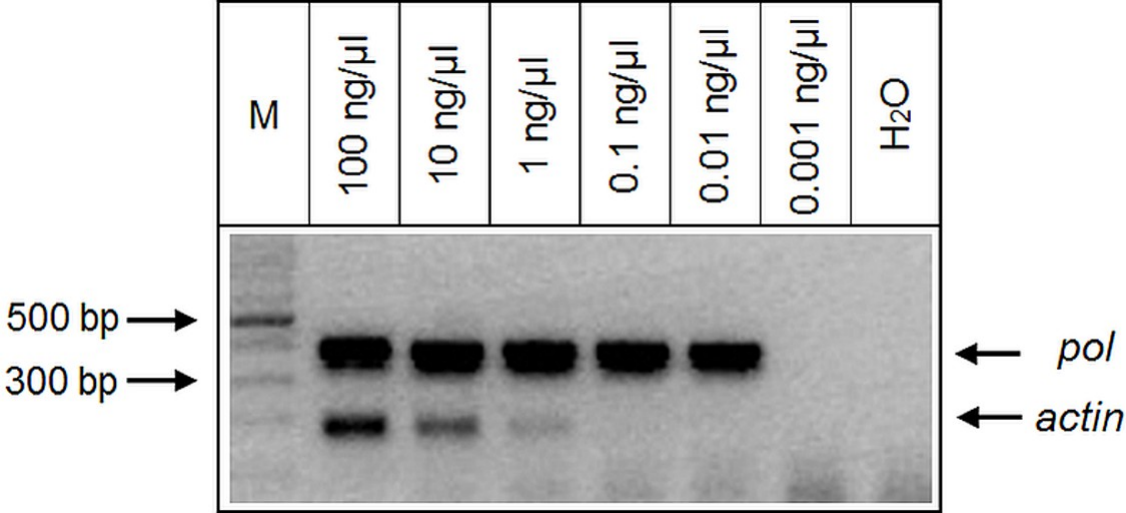
Sensitivity of the multiplex n-PCR. Serial dilutions (1/10) of the DNA obtained from the MT2 cell line, from 100 ng/μl to 0.001 ng/μl, H_2_O (negative control). In each case, the amplification was made with 5ul of DNA from each dilution. The arrows point to the band of *pol* and *actin* amplification and 100 bp DNA Ladder (M).

The sensitivity of the n-PCR was evaluated using serial dilutions (1/10) of the DNA obtained from the MT2 cell line. The *actin* amplification was still detected at the concentration of 1 ng/μl, but not at 0.1 ng/μl of MT2 DNA. In contrast, the *pol* amplification was still detectable at 0.01 ng/μl, but not at 0.001 ng/μl of MT2 DNA (Fig 2).

### Pool of 10 samples in one nPCR

As another modification, we set up the conditions to analyze a pool of 10 samples in a single n-PCR detecting the presence of HTLV-1. As a control, the amplification of *actin* was also performed separately, to verify the correct extraction of DNA.

**Fig 3:**
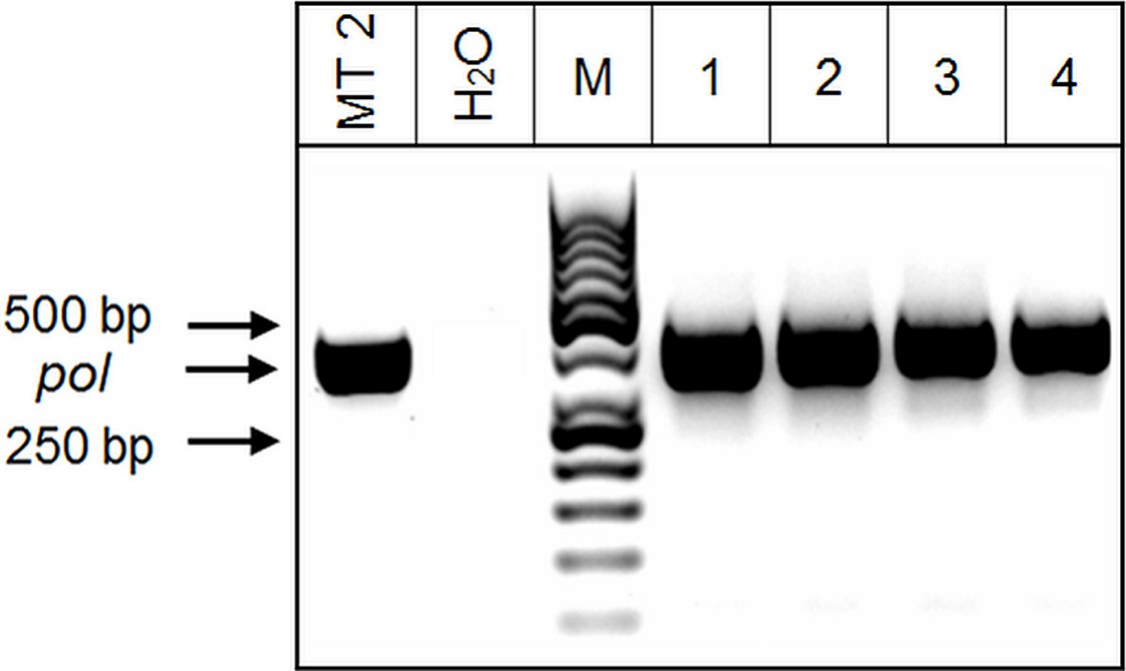
n-PCR for a pool of 10 samples in one PCR. MT2 (positive control), H_2_O (negative control), line 1: HAM/TSP sample, 2: ATLL sample, 3: HAM/TSP sample, 4: HTLV-1 asymptomatic sample. In each case, the amplification was made with 5ul (from 9 non HTLV-1 positive DNA and one HTLV-1 positive). The arrows point to the band of *pol* amplification and the 50 bp DNA Ladder (M).

Each PCR tube contained 18 μl of pooled DNA from 9 negative HTLV-1 samples and one HTLV-1 positive sample. Fig 3 shows the *pol* fragment amplification in MT2 (positive control) and in lines 1–4 (HTLV-1-positive samples).

In subsequent experiments, the sensitivity of the pooled-PCR, was evaluated using serial dilutions (1/10) of MT2 DNA mixed with DNA pooled from 9 negative donors. Under the given experimental conditions, the *pol* amplification was measurable at 0.01 ng/μl, but not at 0.001 ng/μl, of MT2 DNA (data available at https://figshare.com/s/b24ff1bedb99814d4136).

### Samples analysis

To carry the blind study, the samples were selected based on the suspicion of malignancy; the final diagnostic was seen after the nPCR was conducted. A total of 410 samples were analyzed using the n-PCR with *pol* HTLV-1/*actin* co-amplification, four of them were *actin* negative. Table 1 specifies the total of 406 samples from patients with different cancers-diagnostics that were analyzed for the presence of HTLV-1 and resulted *actin* positive.

**Table 1:** List of 406 samples stored at the Pathology Department at Freiburg Uniklinik, Germany during the period 2008–2012, distributed by diagnosis.

Diagnosis	Number	Percentage
Benign samples (noncancerous)	114	28.08
Adenocarcinoma	103	25.37
Malignant melanoma	31	7.64
Undifferentiated carcinoma	3	0.74
Sarcoma	1	0.25
Sarcomatoid carcinoma	1	0.25
Undetermin	34	8.37
B-Cell Lymphom	27	6.65
Abnorme B-Lymphozytose	1	0.25
B-cell lymphoma of marginal zone spleen	2	0.49
NK lymphoma	3	0.74
T lymphoma	51	12.56
Lymphocytosis	2	0.49
Lymphoplasmocytic lymphoma	3	0.74
Leukemia	21	5.17
non-Hodgkin lymphoma	7	1.72
Coexisting hairy cell leukemia and mantle cell lymphoma population	1	0.25
Hair cell leukemia	1	0.25
**TOTAL**	**406**	**100**

**Fig 4:**
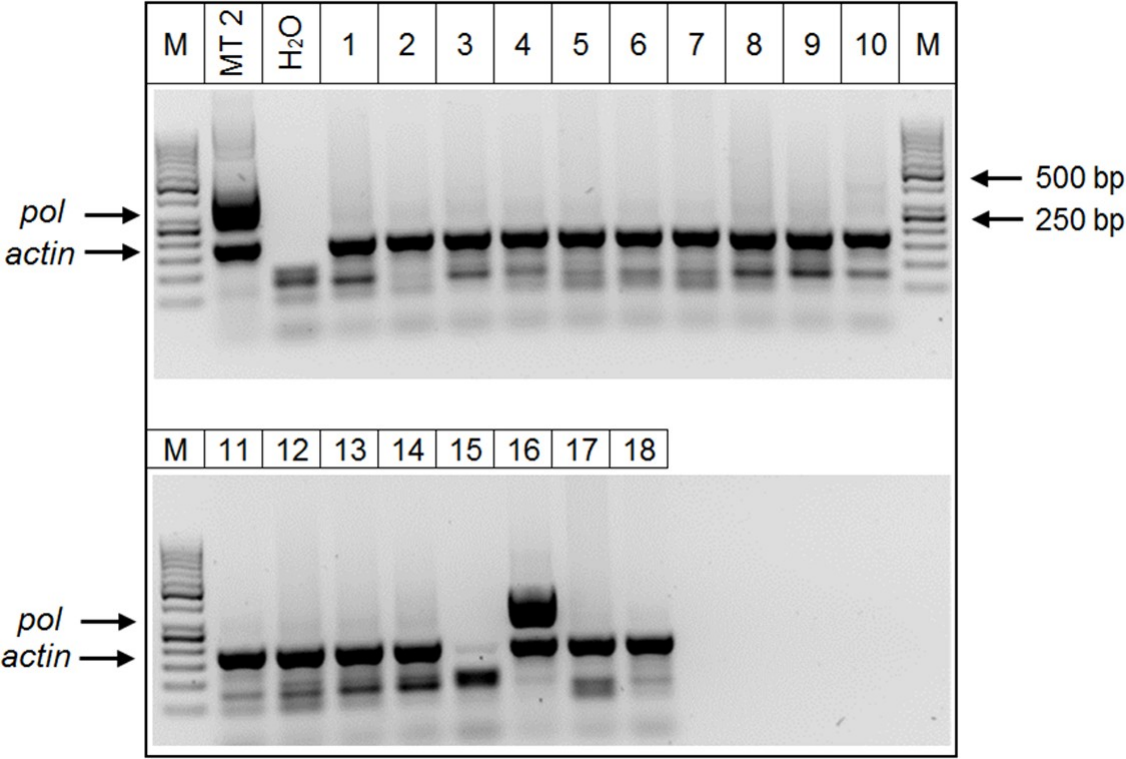
nPCR with *pol* HTLV-1 and *actin* co-amplification. MT2 (positive control), H_2_O (negative control), lines 1–18: samples with suspicion of malignancy. The arrows point to the band of *pol* and *actin* amplification and 50 bp DNA Ladder (M).

**Fig 5:**
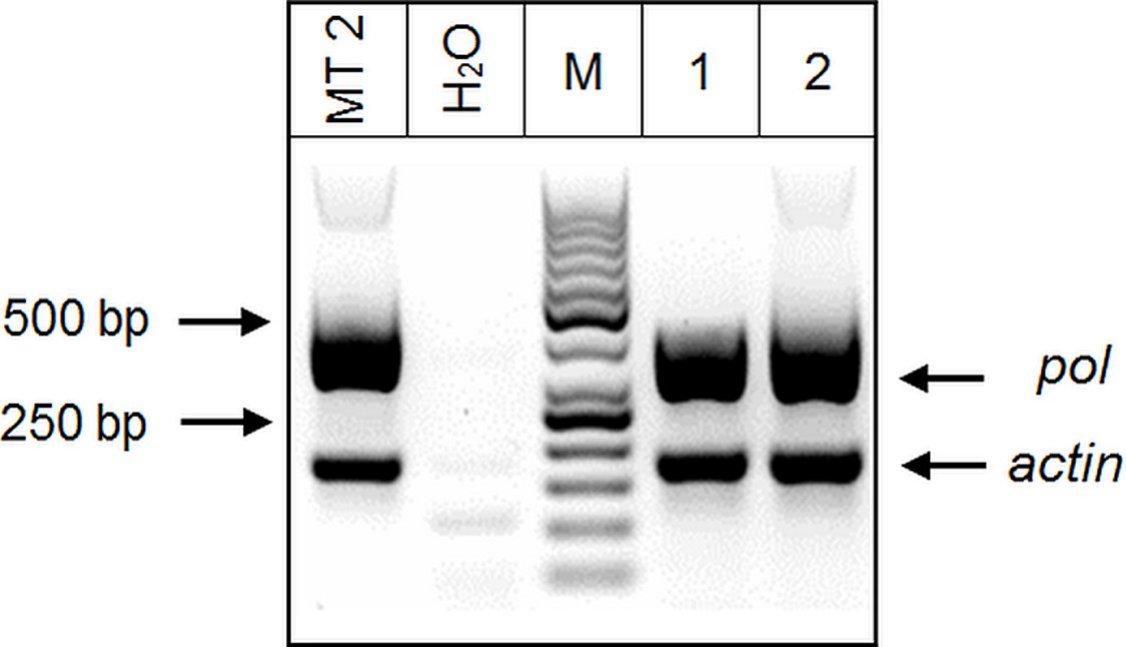
Detection of HTLV-1 *pol* gene by n-PCR from a positive German´s case with T-cell lymphoma. MT2 (positive control), H_2_O (negative control). Samples from German patient: line 1: DNA stored in 2011, obtained from tissue; line 2: DNA stored in 2014, obtained from blood. The arrows point to the band of *pol* and *actin* amplification and 50 bp DNA Ladder (M).

A positive case for HTLV-1 (0.25%) was detected in a sample corresponding to a T-cell lymphoma from 2011. In Fig 4, the *pol* and *actin* amplifications were observed in the positive control sample (MT2) and in the positive case (line 16). The remaining samples in lines 1–14, 17 and 18 showed only the *actin* gene amplification. In line 15, amplification of *actin* was barely detected and it was consider as negative for *actin* amplification. A second molecular diagnosis of this case was performed using the same DNA extracted from tissue in 2011 and another sample of DNA extracted from the peripheral blood in 2014 (Fig 5).

## Discussion

The prevalence of HTLV-1/2 in Germany is low (about 7/100,000) even in drug users and the HTLV-1 screening of blood and organ donors is consequently not mandatory in this country [20–22]. Nonetheless, rare cases of the diseases associated with HTLV-1 (HAM/TSP and ATLL), are occasionally detected [23, 24]. Particularly, in 2013, two transplant recipients from a multi-organ donor were subsequently tested positive for HTLV-1 and developed a cutaneous T-cell lymphoma after transplantation [25].

In this retrospective study, we detected one HTLV-1 positive case among 406 selected samples stored in the Pathology Department at Freiburg UniKlinik during 2008–2012. The HTLV-1 infection was confirmed by the *pol* gene amplification in DNA samples extracted from tissues in 2011 and blood in 2014. The corresponded medical diagnostic for those years are T-cell lymphoma and a HTLV-1 lymphoma, respectively. The prevalence of HTLV-1 in this population was 0.25% (1/406), but if we consider only the patients with lymphocytic disorders the prevalence was 1.4% (1/67). Since this prevalence is relatively high compared to previous reports from Germany, we believe that in order to reach a more accurate picture of the real impact of HTLV-1 infection in this country, more studies need to be conducted.

The implementation of mandatory HTLV-1/2 testing may not be cost-effective, but applying perhaps a more detailed, HTLV-1/2-risk-factors-assessing interview to blood and organ donors should be considered as it has been recommended by the *Public Health—European Commission* [13]. Otherwise, screening for HTLV may be proposed for populations with higher HTLV prevalence such as persons coming from or regularly visiting HTLV endemic areas or areas without HTLV epidemic knowledge. Recently, many immigrants came to Germany from countries with no information about the local HTLV-1/2 situation such as Syria, Kosovo, Albania, Macedonia, Serbia, and probably also from HTLV-1-endemic areas such Iran and Romania [6, 26].

As it was mentioned in the introduction, we believe that molecularly diagnosing HTLV is a logical and economic approach, especially in endemic regions [16–19]. In addition, in countries with very low prevalence, the molecular diagnosis could be performed, for example, in cancer centers where DNA is often extracted for other purposes, for all patients with T-cell malignancies.

A number of reviews and research articles have provided a detailed description of the key parameters that may influence the performance of n-PCR. Fewer publications discuss multiplex PCR [27]. Other studies performed a multiplex PCR for HTLV-1 and -2 but without implementing the n-PCR approach, which could have generated false-negative results [28, 29]. When performing n-PCR techniques for amplifying both the *actin* and the *pol* gene, samples can be analyzed faster, cheaper and with an increased efficacy. Furthermore, we were able to adapt the n-PCR for analyses of 10 samples in one reaction, which makes this *‘pooling n-PCR’* time-saving even though the PCR for *actin* amplification needs to be run separately. In particular, for this study, we considered that the 410 samples selected were a small number and decided to test them only by co-amplification nPCR, and not by doing pools. Nevertheless, we were capable of validating the high sensitivity of this procedure even when analyzing 10 samples at the same time and illustrate its potential to study the HTLV involvement in distinct disorders. Consequently, employing this strategy may a) increase the safety of blood and organ donations, b) diminish the risk of losing donors due to false-positive screening results and c) help establish definitive diagnoses of diseases known to be causatively associated with HTLV-1. In conclusion, this virus is circulating in the population of Germany although the low prevalence, probably due to the greater number of immigrants in recent years. Physicians should bear HTLV-1 infection in mind and suspect it regardless the patient's residence.
